# Let’s prevent diabetes: study protocol for a cluster randomised controlled trial of an educational intervention in a multi-ethnic UK population with screen detected impaired glucose regulation

**DOI:** 10.1186/1475-2840-11-56

**Published:** 2012-05-20

**Authors:** Laura J Gray, Kamlesh Khunti, Sian Williams, Stephanie Goldby, Jacqui Troughton, Thomas Yates, Alastair Gray, Melanie J Davies

**Affiliations:** 1 Department of Health Sciences, University of Leicester, Leicester, UK; 2 Diabetes Research, University Hospitals of Leicester, Leicester, UK; 3 Department of Cardiovascular Sciences, University of Leicester, Leicester, UK; 4 Health Economics Research Centre, Department of Public Health, University of Oxford, Oxford, UK; 5 Department of Cardiovascular Sciences, Leicester Diabetes Centre (Broadleaf), Leicester General Hospital, University of Leicester, Gwendolen Road, Leicester, LE5 4PW, UK

**Keywords:** Type 2 diabetes, Prevention, Impaired glucose regulation, Cluster randomised controlled trial, Screening

## Abstract

**Background:**

The prevention of type 2 diabetes is a globally recognised health care priority, but there is a lack of rigorous research investigating optimal methods of translating diabetes prevention programmes, based on the promotion of a healthy lifestyle, into routine primary care. The aim of the study is to establish whether a pragmatic structured education programme targeting lifestyle and behaviour change in conjunction with motivational maintenance via the telephone can reduce the incidence of type 2 diabetes in people with impaired glucose regulation (a composite of impaired glucose tolerance and/or impaired fasting glucose) identified through a validated risk score screening programme in primary care.

**Design:**

Cluster randomised controlled trial undertaken at the level of primary care practices. Follow-up will be conducted at 12, 24 and 36 months. The primary outcome is the incidence of type 2 diabetes. Secondary outcomes include changes in HbA1c, blood glucose levels, cardiovascular risk, the presence of the Metabolic Syndrome and the cost-effectiveness of the intervention.

**Methods:**

The study consists of screening and intervention phases within 44 general practices coordinated from a single academic research centre. Those at high risk of impaired glucose regulation or type 2 diabetes are identified using a risk score and invited for screening using a 75 g-oral glucose tolerance test. Those with screen detected impaired glucose regulation will be invited to take part in the trial. Practices will be randomised to standard care or the intensive arm. Participants from intensive arm practices will receive a structured education programme with motivational maintenance via the telephone and annual refresher sessions. The study will run from 2009–2014.

**Discussion:**

This study will provide new evidence surrounding the long-term effectiveness of a diabetes prevention programme conducted within routine primary care in the United Kingdom.

**Trial registration:**

Clinicaltrials.gov NCT00677937

## Background

Type 2 diabetes mellitus (T2DM) represents one of the greatest global public health challenges in the 21^st^ century [1]. High glucose levels are currently recognised as the third leading cause of mortality globally and treatment accounts for 7-14% of total health care spending across all global regions [2,3]. International and national health care organisations have responded to this urgent health care need by focusing on recommendations and policy aimed at prevention. In the United Kingdom, this has taken the form of the NHS Health Checks programme which is aimed at screening all individuals between 40 to 75 years of age for vascular and metabolic disease risk and then treating high risk individuals accordingly [4]. Preventing T2DM is one of the fundamental aims of this programme. However in the UK, as in many other parts of the globe, translational research has lagged behind policy change and there has been a lack of diabetes prevention programmes specifically developed for, and evaluated in, routine health care settings.

Although large and well conducted randomised controlled trials have consistently shown that lifestyle interventions can reduce the risk of progressing to T2DM by 30 to 60% in those with impaired glucose tolerance (IGT), an intermediary high risk state between normal glucose regulation and T2DM [5], there is no data from the UK. There also remain important gaps in the evidence when it comes to translating diabetes prevention research into practice [6]. The majority of tested lifestyle intervention studies have used intensive behaviour change strategies relying on multiple and lengthy one-to-one patient contacts which would be unsustainable in a routine health care setting due to cost and infrastructure limitations. Several countries have responded to this limitation by developing, evaluating and implementing diabetes prevention programmes that have been tailored to the needs of their specific health care settings [7]. Although these programmes have varied in context and scope, they have consistently settled on utilising group-based educational programmes as the primary vehicle for promoting behaviour change [8-11]. A recent pilot study in the UK added to these international developments by demonstrating that a 3-hour structured education programme was highly effective at promoting behaviour change, improving glucose regulation and reducing the risk of T2DM in those with IGT at 12 months which were sustained at 24 months [12,13]. In the UK, structured education is already a widely advocated method of promoting self-management strategies and a healthy lifestyle in individuals with T2DM and forms an essential component in integrated diabetes management pathways nationally [14,15]. For example, the established DESMOND programme for individuals with T2DM is delivered nationally and internationally as part of routine care and has been shown to be highly cost-effective [16]. Given the current focus on prevention, there is considerable potential and interest for extending the educator training and quality assurance infrastructure that have accompanied programmes focused on diagnosed chronic disease to the prevention of T2DM. However, this approach needs to be rigorously evaluated when conducted in a primary health care setting.

Another potential limitation when considering the translation of diabetes prevention programmes into “real world” settings is the disconnection between the population used in traditional diabetes prevention programmes and routine clinical practice. Diabetes preventions programmes have typically included individuals on the basis of an oral glucose tolerance test (OGTT) and the presence of IGT [5]. However, such tests may not be appropriate for universal screening given they are costly, time consuming and inconvenient [17]. Both patients and health care professionals have reported that the OGTT is a barrier to attending screening [18]. Additionally studies have shown a low up-take to screening with an OGTT [19] and participation in targeted screening programmes is generally higher [20,21]. Therefore pragmatic alternatives are required. Current international consensus favours a stepped approach whereby high risk individuals are identified using risk score technology which is followed by a blood test to confirm high risk status and rule out the presence of T2DM [22]. Those confirmed with a high risk status can then be referred to a prevention programme. However, the effectiveness and cost-effectiveness of combining a stepped screening strategy with a behaviour intervention has not been evaluated in a usual health care setting; this greatly limits the ability of health care commissioners to make informed decisions when allocating resources. This study will address these points by screening those deemed at high risk of T2DM using a risk score and recruiting those found to have Impaired Glucose Regulation (IGR, a composite of IGT and/or impaired fasting glucose (IFG)) into a prevention programme, and then by formally assessing the cost-effectiveness of this strategy.

## Aim

The aim of the study is to establish whether a pragmatic structured education programme targeting lifestyle and behaviour change in conjunction with motivational maintenance via the telephone is cost-effective and can reduce T2DM incidence in people with IGR identified through a two stage screening programme in primary care.

## Methods/Design

The study consists of two phases. A screening phase where people at risk of IGR/T2DM are identified using a validated risk tool and secondly an intervention phase where those identified with IGR will be recruited in to a T2DM prevention cluster randomised trial.

### Phase 1: the screening phase

#### Identification of those at high risk of having IGR/T2DM

All participating GP practices will receive a Practice Pack giving them general information and contact numbers for the study. All practices will have an induction visit from the project lead and research assistant who will provide training and support.

An automated risk score will be used to identify those at high risk of IGR/T2DM using data routinely stored on individual GP practice computer databases. Various risk scores have been developed and validated for identifying T2DM [23]. Scores available to date have not been validated for the UK multiethnic population and do not additionally pick up those with IGR. Therefore we will use a score developed using data from a previous screening study carried out in Leicester [24]. This score has been amended to take into account ethnicity using the percentage of South Asians within the practice as a proxy for individual ethnicity.

Before the risk tool is applied to a practice database the quality of the data completion is assessed. If the quality level of Body Mass Index (BMI) data recorded is less than 40% practices will be asked to increase this before the risk tool can be used. The score will be calculated for all members of a participating practice. The practice list will then be ranked by risk score with those with the highest scores having the highest risk. The top 10% of patients with the highest score will be invited initially for screening. This 10% limit can be increased to generate further invitations and increase inclusion in the study if required. Where the top 10% of the risk score identifies fewer than 500, all patients within the top 10% will be invited. Where the number of eligible patients identified in the top 10% is greater than 500, the first 500 patients within the top 10% will be invited. If the response rate to initial invitations is insufficient a second mailing of invitations will be conducted. A computer programme will be written to automate the process and produce an excel spreadsheet listing risk scores in descending order.

The invitation will include a patient information sheet and a reply sheet, so patients can register their interest in taking part in the study. A self addressed envelope will be provided for returning of slips. Patients will also be given the number of a dedicated phone line to contact if they are interested and/or require further information. Written informed consent will be taken from all participants and participants will be able to withdraw from the study at any time.

#### Inclusion criteria

Patients are invited for screening if they fulfill the following criteria:

· High risk according to a validated practice risk tool

· Aged 40 to 75 years if English speaking European or 25–75 years if South Asian

#### Exclusion criteria

Patients are excluded from the study if they are/have:

· Unable to give informed consent

· Pregnant or lactating

· Established diabetes

· Terminal illness

· Require an interpreter for language other than South Asian

#### Baseline screening visit

Participants will be asked to fast for 8 hours prior to attending the screening appointment and to bring a list of prescribed medications with them. Before beginning the overnight fast participants are asked to consume their regular evening meal and take any medication as normal. All participants receive a standard 75 g OGTT following informed consent being taken. Those patients who do not wish to have an OGTT will be discontinued from the study and return to their GP for routine care. Plasma samples are obtained immediately before (fasting plasma glucose) and 120 minutes after the glucose challenge (two hours post challenge glucose) along with fasting samples for serum urea and electrolytes, liver function, lipids (total cholesterol, LDL-cholesterol, HDL-cholesterol, triglycerides), and HbA1c. A number of biomarkers will also be measured including: tumor necrosis factor-α, interleukin-6, leptin, adiponectin, resistin, hs-CRP, and PAI-1. Insulin resistance will be measured using HOMA-IR. Levels of vitamin D and C will also be measured.

Results will be relayed via written correspondence and copied to participant and general practitioner. All biochemical measurements will be performed in-house at the University Hospitals of Leicester NHS Trust, UK. Glucose samples will be taken in fluoride oxalate test tubes and placed immediately in a portable 4 litre 4°C refrigerator. HbA1c% will be analysed by a DCCT aligned Biorad Variant HPLC II system (Bio-Rad laboratories, Hemel Hempstead, UK). The imprecision coefficient of variation of this machinery is <0.1%, and the reference intervals fit with national recommendations valid for carriers of variant Hb S, C and Q. Samples will be processed within a maximum of two hours, using an Abbott Aeroset clinical chemistry analyser (Abbott laboratories, Maidenhead, UK), which employs the hexokinase enzymatic method. This machinery has an imprecision coefficient of variation of 1.61%. Serum total cholesterol, HDL-cholesterol, LDL-cholesterol and triglycerides will be measured by means of enzymatic techniques (Dade Behring Dimension analyser, Newark, USA). Plasma creatinine will be analysed with kinetic colorimetric methods. Plasma levels of urea and electrolytes, bilirubin, alanine aminotransferase, alkaline phosphatase and thyroid stimulating hormone will be analysed by means of the Dade Behring Dimension analyser.

Participants will be categorised according to World Health Organisation (WHO) criteria [25]. Diabetes will be defined as a fasting blood glucose of greater or equal to 7 mmoll^-1^ and/or 2 hour plasma glucose of greater than or equal to 11.1 mmoll^-1^. Anyone who has an OGTT result in the diabetes range will be recalled as soon as possible for a second, confirmatory test for diabetes. Those found to have diabetes at baseline will discontinue the study and receive standard diabetes care from their general practitioner; those found to have diabetes during follow up will remain in the study (but not receive OGTTs, further follow up education or support phone calls) and again be referred to their general practitioner for their diabetes care. In this study IGR or ‘pre-diabetes’ will be defined as IFG and/or IGT. IFG will be defined as a fasting blood glucose concentration of between 6.1 and 6.9 mmoll^-1^ inclusive and IGT as a 2-hour blood glucose concentration of between 7.8 and 11 mmoll^-1^ inclusive.

Anthropometric measurements will be performed by trained staff using standard operating procedures. BMI will be calculated after the body weight (kg) and height (m) are measured, weight to be measured in light clothing without shoes to the nearest 0.5 kg. Waist circumference will be measured with a soft tape on standing participants, mid-way between the lowest rib and iliac crest to the nearest 0.1 cm. Hip circumference will be measured over the widest part of the gluteal region, and the waist-to-hip ratio calculated. Three blood pressure recordings will be obtained from the right arm of the patient in a sitting position after 3 minutes of rest, at 1 minute intervals, and then the mean value will be calculated of the second and third reading discounting the first. Seven day step count will be assessed by giving all participants a sealed piezoelectric pedometer (NL-800). Participants will be asked to wear the pedometer, fitted to their trunks (placed on right anterior axillary line) for seven consecutive days during waking hours. Participants will be provided with a stamped addressed envelope to return the pedometers to the study co-ordinators.

A trained nurse will collect data on previous and current medical history, medication and family history using a standard form. Self completed questionnaires will be used to assess smoking status, alcohol consumption, occupation, sleep habits and ethnicity. Social deprivation will be determined by assigning an Index of Multiple Deprivation (IMD) score to participant postcodes [26]. IMD scores are publicly available continuous measures of compound social and material deprivation which are calculated using a variety of data including current income, employment, health, education, and housing.

The following validated questionnaires will also be collected

· The Dietary Instrument for Nutrition Education or DINE food frequency questionnaire will be used to assess dietary fat and fibre intake [27]

· The Health State Descriptive System to assess quality of life, known as 15D [28]

· The Hospital Anxiety and Depression HADS – validated for depression and anxiety relating to diagnosis of condition and the care provided thereafter [29]

· The Brief Illness Perception Questionnaire or BIPQ designed to quickly assess cognitive and emotional representations of illness [30]

· The International Physical Activity Questionnaire (short form) (IPAQ) to obtain internationally comparable data on health–related physical activity [31]

Patients will also self-report on two questions concerning sleeping pattern (how many hours sleep did you get last night? And on average, how many hours do you sleep in 24 hours) [32].

#### Outcomes

The primary outcome of the screening phase is the proportion of people detected with IGR or T2DM using a validated risk tool (positive predictive value). Secondary outcomes include the response rate to the invitation to screening. Those with IGR will be asked if they would like to take part in phase 2 – the diabetes prevention cluster randomised controlled trial.

### Phase 2: Cluster randomised trial

Phase 2 is designed to adhere to internationally recognised criteria for developing complex interventions and for undertaking and reporting cluster randomised controlled trials [33]. Randomisation will be conducted at the level of the GP practice by a researcher who is independent of the study team. Cluster designs are being used in other similar trials of lifestyle management interventions [34-36]. Practices will be randomised 1:1 to either the control arm or the intensive arm using stratification by list size (<6,000, ≥6,000), and ethnicity (% South Asian <21%, ≥21% (median level of % South Asian in the ADDITION-Leicester study [37]) with a block size of 4.

**Figure 1:**
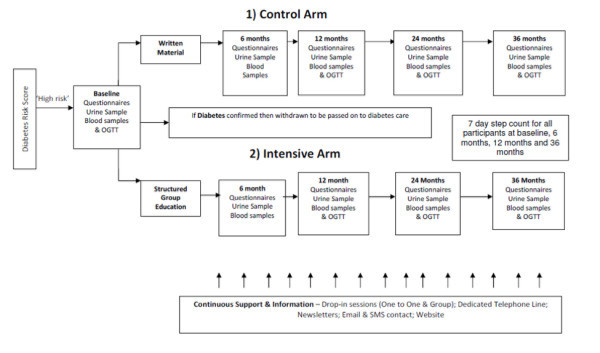
Study participation.

Participation in the study is summarised in Figure 1. A summary of the data collected at each time point is given in Table 1. The primary outcome data will be collected at 36 months. Additional data will be collected at 6, 12 and 24 months. The data collection will follow the same standard operating procedures as the baseline/screening data described in Phase 1. Additionally at the 3 year follow up a health resource one page questionnaire and the EQ-5D will be collected [38]. Records are kept on missed clinical visits; structured education programmes and telephone support as well as withdrawn subjects.

**Table 1:** Clinical assessment and measures

Measurements	Time points				
	Baseline/Screening	Trial 6 m	Trial 12 m	Trial 24 m	Trial 36 m
	Clinical Assessment					
Medical History	X		X	X	X	
Medication History	X		X	X	X	
Physical Exam	X		X	X	X	
Cardiovascular Risk Score	X	X	X	X	X	
Presence of Metabolic Syndrome	X	X	X	X	X	
	Anthropometric					
3 x Blood Pressure	X	X	X	X	X	
Height	X				X	
Weight	X	X	X	X	X	
Waist Circumference	X	X	X	X	X	
Blood Tests						
Oral Glucose Tolerance Test	X		X	X	X	
HbA1c	X	X	X	X	X	
Lipids	X	X	X	X	X	
Urea & Electrolytes	X		X	X	X	
Liver Function Tests	X		X	X	X	
	Questionnaires & Lifestyle Measures					
IPAQ – SF [31]	X	X	X	X	X	
DINE [27]	X	X	X	X	X	
BIPQ [30]	X	X	X	X	X	
HADS [29]	X	X	X	X	X	
15D [28]	X	X	X	X	X	
	Health care resource use					X
EQ-5D [38]					X	
Sleep questions	X	X	X	X	X	
7 Day Step Count	X	X	X	X	X	
Urine Sample	X		X	X	X	

#### Inclusion criteria

Patients who were eligible for phase 1 are included in the trial if

· Diagnosed with IGR (IGT and/or IFG) at the OGTT at screening/baseline visit

#### Exclusion criteria

Patients are excluded from the trial if they are:

· Diagnosis of diabetes at screening/baseline (if diagnosed during the study participants are invited to continue without the 120 glucose sample or further intervention activities)

#### Control arm intervention

Control subjects receive a booklet detailing information on risk factors for T2DM and how physical activity and lifestyle change can be used to prevent or delay the disease. The leaflet addresses factors around T2DM risk using the five domains (causes, consequences, identity, control/treatment, and timeline) highlighted by Leventhal’s common sense model [39]. The follow up sessions for the control group will occur at the same time points as the intervention group and the same data will be collected.

#### Intensive arm intervention

Participants in this arm receive the same information booklet as the control arm and in addition are invited to attend an initial six hour structured education programme called Lets Prevent (LP), three monthly nursing support phone calls, and a yearly three hour update structured education programme.

The style, content and process of the programme draws on a range of concepts from health psychology and education [39-42] and its philosophy is centred on patient empowerment [43]. In short, it is a six hour group based education programme that can be either delivered in one full six hour day or in two three hour sessions. The programme has a written curriculum, an outline of which can be seen in Table 2. The key food messages are taken from the Diabetes Prevention Program [44] and the Finnish Prevention Study [45]. Goals are to attain a sustained weight reduction of greater than 5% body weight, moderate reduction in total fat of less than 30% energy intake, low saturated fat intake of less than 10% energy intake and higher fibre intakes of greater than 15 g per 1,000 calories.

**Table 2:** The Lets Prevent curriculum content

Session 1	Theory	Sample Activity	Duration
Introduction	-	-	10 mins
Patient story	CSM	Participants asked to tell their story about how they discovered they had pre diabetes and their current knowledge of pre diabetes	30 mins
Professional story	CSM, DPT	Uses participants’ stories to support them in learning how the body regulates glucose	50 mins
Taking control 1 Weight management	CSM, DPT, SLT	Uses participants’ stories to support them in discovering how weight/waist affects pre diabetes. Provides knowledge and skills for food choices to control weight	30 mins
Physical activity	CSM, DPT, SLT	Uses participants’ stories to support them in discovering how physical activity affects pre diabetes. Provides knowledge and skills for activity choices to manage pre diabetes	40 mins
How am I doing?	SLT	Participants reflect on what issues have come up in the programmes so far	5 mins
Session 2	Theory	Sample Activity	Duration
Reflections	SLT	Participants reflect issues that have arisen in the programme so far	10 mins
Professional story	CSM	Uses participants’ stories to support them in discovering how other risk factors (e.g. blood pressure and cholesterol) affect pre diabetes and the development of complications	30 mins
Taking control 2 Food choices: focus on fats	DPT, SLT	Provides knowledge and skills for food choices to reduce risk factors	50 mins
Self management plan	SLT	Participants supported in developing their self management plans	30 mins
Questions	CSM	Checks that all questions raised by participants throughout the programme have been answered and understood	40 mins
What happens next?	SLT	Follow up care outlines	5 mins

CSM: Common sense model, DPT: Dual processing theory, SLT: Social learning theory.

The physical activity messages are taken from the Pre-diabetes Risk Education and Physical activity Recommendation and Encouragement Programme (PREPARE) [12,13]. The PREPARE programme successfully demonstrates that a pragmatic education programme that incorporates pedometer use is effective in improving glucose tolerance in those with IGT. As in the PREPARE programme, participants are provided with a pedometer as a tool for promoting self-regulatory strategies such as goal setting and self-monitoring. The physical activity goal is to achieve an increase in daily walking of 45 minutes or 4,500 steps. Goal attainment is encouraged through the use of proximal objectives such as increasing steps by 500 per day every two weeks.

Following 12 and 24 month clinic appointments, participants are offered the option of attending a three hour update session. The purpose of this update session is to review key messages, review personal risk and action plan. Throughout the three year intervention, participants receive three monthly telephone contacts from nurses trained to support participants with their chosen behaviour change. A quality development programme ensures that the educational intervention is delivered in such a way that the core content and learning outcomes are achieved and the educator behaviours are linked to the programme philosophy and learning theories. The quality development programme consists of internal and external processes adapted from findings from the DESMOND collaborative [46].

Methodologies previously used to effectively modify the DESMOND module to be suitable for those from Black and Minority Ethnic (BME) groups were used to develop the LP BME intervention [47]. The core educational messages are the same as in LP but the programme is culturally appropriate and non reliant on the written word. The LP BME programme is delivered as four sessions of three hours if delivered with an interpreter or two three hour sessions without an interpreter.

#### Endpoints and outcomes

The primary outcome aims to show the reduction in the incidence of T2DM at 36 months in people with screen-detected IGR. Secondary outcomes are reductions in HBA1c, blood glucose levels fasting and post glucose load, cardiovascular risk as calculated by the Framingham risk calculator [48] and the presence of Metabolic Syndrome as defined by NCEP ATP III [49], increasing seven day step count and cost-effectiveness of the intervention.

#### Power calculation

Assuming a 3 year cumulative conversion rate to T2DM of 35% in the control group [44,45,50], an intra class correlation of 0.05 and a dropout rate of 20% (as seen in the Finnish Prevention Study [51]), we calculated that we would need 374 patients to consent per group to detect a 40% risk reduction in the intervention group – data from 44 practices, 17 participants per practice, with 80% power at the 5% significance level. For 17 participants to be recruited per practice we have assumed a participation rate of around 20%. For an average practice 500 people will be invited for screening, of these around 20% will have pre-diabetes (assuming a positive predictive value of 20% [24]). Therefore around 100 participants per practice will be eligible for the trial. Assuming a participation rate of 20% should give the 17 participants we need per practice. A participation rate of 20% has been seen in other studies in a similar population [19].

A 40% reduction in the relative risk of developing T2DM was chosen as a conservative interpretation of the current evidence. Several meta-analyses and a systematic review have shown that lifestyle intervention studies in those with IGT resulted in a ~50% reduction in the relative risk of developing T2DM [5,52,53]. An intention to treat analysis of 12-month follow-up data from a randomised controlled trial found that an intervention aimed at the promotion of physical activity, using methods that are similar to the proposed study, achieved a reduction in 2-hour glucose of 1.3 mmol/l compared to the control group [12]. A reduction of 2-hour glucose of this magnitude has been associated with around a 50% reduction in the relative risk of developing T2DM [52], which is consistent with the above evidence.

One secondary outcome will be the percentage of patients in each group with a 10-year CVD risk greater than 20% at end of 3 years. It is estimated that 55% of patients will have a CV risk greater than 20%. To detect a difference between the two groups of 20% points in the proportion of patients with a 10 year risk of >20% with 80% power and two alpha of 5% and an intra-practice correlation coefficient of 0.05 the required sample size is 180 in the two groups.

#### Data analysis

At major time points and at study completion the findings will be reported according to the internationally recognised CONSORT statement for the reporting of cluster randomised control trials [54]. Data will be analysed on an intention to treat basis (ITT). Data will be analysed using STATA v10, and all analysis will take into account the clustering by GP practice. Survival curves would be calculated to estimate the cumulative incidence of diabetes. The difference in incidence of T2DM in the groups is tested using the two-sided log-rank test adjusted for cluster. Differences in secondary outcomes between the groups will be assessed using either linear for continuous outcomes or logistic for categorical outcomes regression with treatment group as the independent variable.

#### Health economics

An economic evaluation will be conducted alongside the study. The objective will be to estimate the cost-effectiveness of the educational intervention compared to control. Resource use, costs and health outcomes will be measured in each arm of the study, and cost-effectiveness will be calculated as the difference in costs divided by the difference in effects. Costs will include the costs of the initial and ongoing intervention, drug use, all health care consultations and visits, and hospitalisations. These will be collected directly from the participants using the using from trial case record forms (for medication data and hospitalisations) and a simple (one-page) questionnaire given to all patients at the final (36-month) visit, which will also record patient incurred costs. Effectiveness will be measured as 1) incidence of T2DM at 36 months, in line with the primary outcomes, and 2) quality adjusted life years gained. Within-trial quality of life will be measured using the 15D instrument at baseline and all follow-up points, and the EQ-5D at the final visit [38]. Long-term cost-effectiveness will be estimated by extrapolating from 36 months using the Framingham risk equation. Predictions for patients with diagnosed diabetes will be cross-checked using the UKPDS Outcomes Model. Uncertainty around all estimates will be fully reported using recommended parametric and non-parametric methods, with additional sensitivity analyses for areas of methodological or policy uncertainty. If the intervention appears cost-effective in the trial comparison, the likely screening costs in normal practice (rather than the screening costs in the trial, which may include protocol-driven elements) will also be estimated and included. We will undertake a subsidiary analysis on differences by allocation in changes in employment hours, nature or status using self reported data collected at baseline, 6 months and the annual follow ups.

### Funding and timescale

The project is funded by an NIHR Programme Grant. Screening started in July 2009 and the last patient should attend their last follow up in July 2014.

## Discussion

To our knowledge this will be the first study in the United Kingdom to establish the long-term effectiveness of an intervention structured education programme to promote lifestyle change in those with a high risk of T2DM identified using a risk score. This complete programme of screening for high risk individuals followed by a lifestyle modification intervention is in line with the recommendations from the IMAGE project [55]. Whilst both the effectiveness and cost-effectiveness of structured education programmes at preventing T2DM has been established, there has been a lack of translational research aimed at patients at risk and/or with IGR, specifically designed for a routine health care setting in the UK. This study will help address this limitation and aims provide an effective solution to this need.

The Let’s Prevent programme was specifically designed for translation into routine care. Structured education is already recommended by NICE for promoting a healthy lifestyle and self-management in those with diagnosed T2DM and has a track record of implementation and delivery in usual health care practice spanning the last decade. For example, the DESMOND programme, on which the structure and underlying philosophy of Let’s Prevent is based, is delivered in over half of all primary care organisations nationally and is supported by an established infrastructure for training and quality assuring educators at a national level [46,56]. Let’s Prevent was designed to be suitable for integration into these types of existing platforms for the delivery of structured education in primary care. This will allow primary care organisations to commission a suite of structured education programmes to meet the needs of their diabetes pathways. This is particularly relevant to current health care policy in the United Kingdom, where the prevention of diabetes and other chronic diseases is actively targeted and supported through the NHS Health Checks Programme [57]. NICE have also recently drafted guidance around the prevention of diabetes [58]. These policies and recommendations advocate the use lifestyle interventions for high risk patients as the central pillar of any diabetes prevention pathway. Therefore, it is important that commissioning groups have access to intervention programmes that have been rigorously evaluated for effectiveness and cost-effectiveness and which are suitable for direct translation into a routine primary care setting.

## Abbreviations

T2DM: Type 2 diabetes; IGT: Impaired glucose tolerance; IGR: Impaired glucose regulation; IFG: Impaired fasting glucose; WHO: World health organization; OGTT: Oral glucose tolerance test; BMI: Body mass index; IMD: Index of multiple deprivation; HADS: Hospital anxiety and depression scale; BIPQ: Brief illness perception questionnaire; IPAQ: International physical activity questionnaire; LP: Let’s prevent; PREPARE: Pre-diabetes risk education and physical activity recommendation and encouragement programme; BME: Black and minority ethnicity; ITT: Intention to treat.

## Let’s prevent collaborators

A Farooqi, M Carey, K Abrams, T Skinner, J Tuomilehto, S Heller, N Samani, B Stribling, K Jones.

## Competing interests

The author’s declared that they have no competing interests.

## Authors’ contributions

LJG drafted the manuscript, participated in the design of the study, carried out the sample size calculation. KK Contributed to the conception and design. SW participated in the acquisition of data, SG participated in the acquisition of data, JT participated in the design of the study and the intervention, TY participated in the design of the study and the intervention, AG participated in the design of the study with regards the health economic analysis, MJD Contributed to the conception and design. All authors read and approved the final manuscript.
